# Virtual versus real cystoscopy

**Published:** 2007

**Authors:** Rajiv Yadav, Rajeev Kumar

**Affiliations:** Department of Urology, All India Institute of Medical Sciences, New Delhi, India. E-mail: rajeev02@gmail.com

## SUMMARY

Forty-seven consecutive patients (28 men and 19 women, age range, 35-72 years) under evaluation for painless gross hematuria were prospectively studied. Unenhanced followed by contrast enhanced CT images were taken (detector array, 4 × 5 mm; beam pitch, 1.5) on a 4-MDCT scanner (Multidetector CT, Light Speed QX/I, GE Healthcare). Delayed scanning dedicated for bladder evaluation was also performed after the patient had sensation of bladder fullness. Adequate mixing of contrast and urine in the bladder was ensured by postural changes prior to acquisition of delayed bladder scans. The obtained data was subsequently processed for reconstructing virtual cystoscopy (by volume-rendering algorithm) and multiplanar images reconstruction (transverse, coronal and sagittal planes at a slice thickness of 1 mm). Positive findings on imaging were noted as any polypoidal lesion, surface irregularity or focal wall thickening. In order to systemically indicate the location of lesion, the bladder was divided into six sites including anterior, posterior, superior, inferior, right and left sites. Conventional cystoscopy was performed by urologist within a week after CT. The image findings on virtual cystoscopy and multiplanar reconstructed images were independently evaluated by two radiologists blinded to the findings of conventional cystoscopy. Results of the source CT images and reconstruction techniques were compared with the gold standard i.e. conventional cystoscopy using the McNemar test. Agreement between the two observers was evaluated using kappa statistics.

A total of 40 bladder sites in 25/47 (53%) patients had lesions on conventional cystoscopy. Fifteen patients had a single positive site, six had two positive sites, three had three positive sites and one had four positive sites. The interobserver agreement was excellent for virtual cystoscopy, fair for multiplanar reconstruction and good for source CT images. For both observers, the sensitivity for lesion detection by bladder site was significantly greater with virtual cystoscopy (Observer 1, 95%; Observer 2, 90%) than with multiplanar reconstruction (78% and 60%) and source CT (68% and 65%) images (*P* < 0.05), whereas the specificity by bladder site and the sensitivity and specificity by patient did not differ with the three methods (*P* >0.05). For determining the presence or absence of lesion at each site, virtual cystoscopy was more accurate than multiplanar reconstruction and source CT images for both observers (*P* < 0.05).

## COMMENTS

Urinary bladder is an appropriate organ for virtual endoscopy because of its simple luminal morphology, its relatively small volume and the absence of involuntary peristalsis. Since its introduction, virtual cystoscopy has been evaluated by various investigators for the detection of bladder tumor. Virtual cystoscopy examination requires dedicated training and rapid data acquisition which although made possible by advances in software, takes time and significant effort to obtain data for 3D display. Through this study, the authors have compared the diagnostic accuracy of virtual cystoscopy with 2D display of the mucosal surface of the bladder, such as source CT images or multiplanar reconstruction, in the detection of bladder lesions in order to determine its efficacy vis-à-vis the time and effort spent in postprocessing. While virtual cystoscopy proved to be more accurate than multiplanar reconstruction and source CT images for determining the presence or absence of lesion at each site (*P* < 0.05), the sensitivity and specificity for identifying patient with lesions did not differ significantly with the three methods (*P* > 0.05). The sensitivity and specificity for identifying patients with a bladder lesion with virtual cystoscopy were 96% and 86%, respectively.

Despite the obvious benefits of virtual cystoscopy over conventional cystoscopy in terms of less invasiveness and more comfort, it has several limitations. These include low detection rate for lesions smaller than 0.5 cm (up to 60%),[1] difficulty of detecting mucosal and sessile lesions,[2] pseudolesions due to bladder wall distortion in situations like bladder outlet obstruction, previous bladder surgery, radiation or biopsy. Some authors have reported that lesions as small as 3 mm can be detected on virtual cystoscopy.[3] However, the study design in these series was to retrospectively evaluate bladder lesions that had been already confirmed on conventional cystoscopy. Thus although virtual cystoscopy with MDCT may be somewhat better than axial scans and multiplanar reconstructed images, the limitations in clinical practice are too many for this technique to replace conventional cystoscopy.

## References

[1] Song JH, Francis IR, Platt JF, Cohan RH, Mohsin J, Kielb SJ (2001). Bladder tumor detection at virtual cystoscopy. Radiology.

[2] Merkle EM, Wunderlich A, Aschoff AJ, Rilinger N, Gorich J, Bachor R (1998). Virtual cystoscopy based on helical CT scan datasets: Perspectives and limitations. Br J Radiol.

[3] Fenlon HM, Bell TV, Ahari HK, Hussain S (1997). Virtual cystoscopy: Early clinical experience. Radiology.

